# Relationships between internet use, deliberate self-harm, and happiness in adolescents: A Taiwan birth cohort pilot study

**DOI:** 10.1371/journal.pone.0235834

**Published:** 2020-07-10

**Authors:** For-Wey Lung, Bih-Ching Shu, Tung-Liang Chiang, Shio-Jean Lin

**Affiliations:** 1 Calo Psychiatric Center, Pingtung County, Taiwan; 2 Graduate Institute of Medical Science, National Defense Medical Center, Taipei, Taiwan; 3 Department of Nursing, Institute of Allied Health Sciences, College of Medicine, National Cheng Kung University, Tainan, Taiwan; 4 Institute of Health Policy and Management, College of Public Health, National Taiwan University, Taipei, Taiwan; 5 Genetic Counseling Center, Chi Mei Medical Center, Tainan, Taiwan; University of Pennsylvania, UNITED STATES

## Abstract

The potential risk of internet use on adolescents’ self-harm is a major concern. Vulnerable adolescents who are susceptible to bullying are also susceptible to the negative influence of the internet. In this study, the pathway associations were investigated between the risk factors of deliberate self-harm, experience of being bullied, internet use, and protective factors of maternal monitoring on perceived happiness of 12- and 13-year-old adolescents in the Taiwan Birth Cohort Pilot Study dataset (n = 1,457). The Chinese Oxford Happiness Questionnaire was used to measure the adolescents’ self-perceived levels of happiness, in two dimensions of social adaptation status and psychological well-being. Our results show that 354 (24.3%) of the 12-year-olds reported having been bullied, and 289 (19.8%) of the 13-year-olds reported this. Seventy-nine (5.4%) of 13-year-olds reported deliberate self-harm in the past year. Results of a structural equation model showed that those who had been bullied at age 12 years were at greater risk of deliberate self-harm at age 13 years. A negative association was found between duration of internet use and perceived level of happiness. Adolescents who spent >5 h online during days off school were at higher risk of deliberate self-harm, and perceived a lower level of happiness. Therefore, spending >5 h online during days off school could be used as an indicator in future preventive action programs to screen out those at a high risk of excessive internet use, deliberate self-harm, and psychological well-being and social adjustment issues.

## Introduction

The potential influence of internet use on adolescents’ self-harm and suicidal behaviors is a matter of great concern. Because adolescents are at a life stage with developmental issues and experience of stressful circumstances, they are more prone to deliberate self-harm. Self-harm is most frequent in adolescents, especially girls, aged 13–15 years, and also usually begins in this age group [1]. Daine et al. systematically reviewed the influence of internet use on self-harm, and found both positive and negative results [2]. Although the internet can provide support and social networks to adolescents who may be socially isolated, it can also provide access to suicidal content, normalizing self-harm on online forums, and reducing the threshold for self-harm [2]. The influence of internet use on deliberate self-harm is of great concern, given that up to 19.8% of adolescents in Taiwan are considered to have internet addiction [3].

Vulnerable adolescents who are susceptible to social exclusion and bullying are also susceptible to the negative influence of the internet. Adolescents who have been bullied may connect to the internet, because people with low self-esteem are more likely to go online to seek social support, modify their mood, and decrease their loneliness [4]. Isolated and susceptible individuals who are addicted to online activity and visit pro-suicide websites are at high risk of suicidal behavior [2]. However, even without the influence of internet, those who have been bullied are already at higher risk of suicidal ideation and attempt [5, 6].

Apart from the influence on deliberate self-harm, excessive and pathological internet use can also impair psychological well-being, academic performance, and peer and family interaction [7]. However, families with high levels of conflict and dysfunction also constitute a risk factor for internet addiction [8]. In contrast, parental care and protection can also serve as protective factors against internet addiction [9]. Regular exercise has also been found to be a protective factor against excessive internet use, because it can enhance mood and psychological well-being [10].

Happiness is a subjective experience of well-being that can be best reported by the subject. Similar to the potential effect of internet use on deliberate self-harm, internet use can also have positive and negative effects on subjective level of happiness [11]. Virtual relationships can be more intimate than in-person relationships, especially for those who are shy, anxious, and lack face-to-face social skills [12]. However, another study showed that extensive use of the internet was associated with a decline in communication with family members and size of social circle, and escalation of loneliness and depression [13]. Besides internet use, adolescents who have been bullied also perceive lower levels of happiness [14].

Internet use as a potential risk factor for deliberate self-harm, and its association with having been bullied and level of happiness have been investigated separately in previous studies. However, no research has investigated the mediating and moderating association of these factors together, nor have they been investigated in a follow-up birth cohort study. In Taiwan, the 12- and 13-year age groups are in the transitional period from elementary to middle school. In view of the importance of this group, in this study the pathway association was investigated between the risk factors of deliberate self-harm, experience of being bullied, internet use, and protective factors of maternal monitoring on the perceived happiness of 12- and 13-year-old adolescents, using a national birth cohort dataset.

## Materials and methods

### Participants

The Taiwan Birth Cohort Pilot Study (TBCS-P) 12- and 13-year-old dataset was used in this study. The aim of the TBCS-P is to build a sample representative of children in Taiwan. Babies born in November and December 2003 were randomly selected, with no exclusion criteria, using the method of household probability sampling [15]. The primary sampling unit of cities and towns was used at the first stage of a two-stage stratified random sampling [15]. By grouping all 369 municipal areas in Taiwan into 12 strata according to four sizes of towns and three levels of total fertility rate, 85 towns/cities were randomly selected. In the second stage, newborns were proportionally selected according to the rate of birth from the 85 selected towns/cities [15]. A final total of 2,048 babies were selected when the children were aged 6 months (selection rate of 9.51%) [15]. This stage used the dataset of when the children were aged 12 years, 1,561 were followed up, and when the children were aged 13 years, 1,457 were followed up (follow-up rate of 93.34%). Adolescents in these two age groups were included in this study, resulting in a total of 1,457.

All the procedures in studies involving human participants were performed in accordance with the ethical standards of the institutional and/or national research committee and according to the 1964 Helsinki Declaration and its later amendments or comparable ethical standards. The study was approved by the Research Ethics Committee of National Taiwan University Hospital in Taipei, Taiwan (NTUH-REC No. 201701106RINC). A trained researcher visited the homes of the participating families after the parents had agreed to participate. Written informed consent was obtained from the participants and their parents. The researchers then asked the parents and adolescents a set of questions from a structured interview booklet. Data were analyzed anonymously.

### Measures

In the TBCS-P, information was collected from parents’ and adolescents’ self-reports. All factors analyzed in this study were obtained from adolescents’ self-reports. The experience of being bullied and perceived level of happiness were collected at age 12 and 13 years. Internet use, deliberate self-harm, and time spent with friends were collected at age 13 years.

#### Maternal monitoring

Maternal monitoring was measured by asking the adolescents three questions: (1) “Does your mother (or the person who mainly takes care of you) know what you do in your free time?” (2) “Does your mother (or the person who mainly takes care of you) know who you normally socialize with (e.g., when you go out to play, exercise, go shopping or do homework?)”. (3) “Does your mother (or the person who mainly takes care of you) know when you go to bed?”. These items were answered as “always”, “often”, “sometimes”, “once in a while”, or “never”. The ratings of these items were combined to form the maternal monitoring factor, which had a Cronbach’s α of 0.731.

#### Internet use

The number of hours adolescents spent online on computer and smartphone devices during schooldays and days off was determined by asking the adolescents: (1) “How many hours do you spend online during schooldays?”; and (2) “How many hours do you spend online on days when you do not have to go to school?” As well as the continuous variable, the hours adolescents spent online were grouped into those who spent >5 h/day online, and those who spent < 5 h/day. In a previous study it was reported that adolescents who spent >5 h/day on smartphones, compared to those who spent <5 h/day online, had a 66% higher risk of suicide [10].

#### Friends

The factor of spending time with friends was measured by asking adolescents “Do you often play or do your homework with friends?” to differentiate from cyber-friends. This question was answered “always”, “often”, “sometimes”, “once in a while”, or “never”.

#### Bullying and deliberate self-harm

The factors of experience of being bullied and deliberate self-harm were measured by asking the teenagers: (1) “Have you ever been bullied by your classmates?”; and (2) “Have you (intentionally) inflicted injury on yourself in the past year”. Answers were dichotomized into “yes” as 1, and “no” as 0.

#### Happiness

The seven-item Chinese Oxford Happiness Questionnaire was used to measure adolescents’ self-perceived level of happiness, in two dimensions of social adaptation status (SAS) and psychological well-being (PWB) at age 12 and 13 years. The Chinese Oxford Happiness Questionnaire was translated and culturally modified from the English-language version developed by Hills and Argyle [16], and has shown good psychometric properties in adolescents in Taiwan [17]. Items in the PWB dimension included reversed items of: “I do not feel particularly pleased with the way I am” and “I do not have particularly happy memories of the past”; and SAS questions of: “I feel that life is very rewarding” and “I am well satisfied about everything in my life”. The PWB dimension of the Chinese Oxford Happiness Questionnaire resulted in a Cronbach’s α of 0.573, and SAS dimension of 0.765 at age 12 years, and 0.571 and 0.764, respectively, at age 13 years.

### Statistical analysis

Missing data were replaced using Bayesian analysis; a multiple imputation method based on item response theory that accounts for multiple sources of correlation to replace missing data [18]. The demographic distribution of the adolescents and parents was analyzed. Linear regression was used to investigate factors associated with perceived level of happiness at age 13. All analyses were processed using SPSS for Windows version 20.0 (SPSS Inc., Chicago, IL, USA).

The pathway relationship of internet use, experience of being bullied, and perceived level of happiness of adolescents was analyzed using a structural equation model (SEM). The SEM was processed using the Analysis of a MOment Structures 7.0 statistical software package (SPSS). The bootstrapping provided by Analysis of a MOment Structures was used to estimate confidence intervals for all tests of parameters in our models. Furthermore, the models presented were parsimonious, which means that only statistically significant pathways were presented, and all paths with p > 0.05 were deleted from the SEM.

## Results

Results of the descriptive analysis are shown in Table 1. Out of the 1,457 adolescents, 354 (24.3%) reported having been bullied at age 12 years, and 289 (19.8%) reported this at age 13. Seventy-nine (5.4%) adolescents reported deliberate self-harm in the previous year. Regarding internet use, the average time spent online during schooldays was 1.48 h (SD = 1.82), and 4.01 h on days off (SD = 3.28).

**Table 1:** Descriptive analysis of the adolescents (n = 1,457).

Variable	n (%)
Sex	
Boy	791 (54.3)
Girl	666 (45.7)
Bullied at 12 yr	354 (24.3)
Bullied at 13 yr	289 (19.8)
Deliberate self-harm, age 13 yr	79 (5.4)
Play/homework with friends, age 13 yr	
Never	101 (6.9)
Once in a while	276 (18.9)
Sometimes	339 (23.3)
Often	372 (25.5)
Always	369 (25.3)
≥5 h/d online in school days, age 13 yr	87 (6.0)
≥5 h/d online in days off school, age 13 yr	476 (32.7)
Variable (range)	Mean (SD)
Hours spent online, age 13 yr	
School days (h)	1.48 (1.82)
Days off school (h)	4.01 (3.28)

Linear regression was used to investigate factors associated with the level of happiness, including SAS and PWB, at age 13 years (Table 2). Boys had better SAS than girls (β = −0.06, p = 0.006), and SAS at age 12 years was predictive of SAS at age 13 years (β = 0.47, p < 0.001). Interaction with friends increased the level of SAS (β = 0.10, p < 0.001), but deliberate self-harm and increased use of internet on days off school lowered SAS (β = − 0.07, p = 0.001; β = −0.07, p = 0.003). In contrast, both SAS and PWB at age 12 years were predictive of PWB at age 13 years (β = 0.08, p < 0.001; β = 0.38, p < 0.001). Similar to the results for SAS, interaction with friends increased the level of PWB (β = 0.08, p = 0.001), but deliberate self-harm and increased use of internet on days off school lowered PWB (β = −0.14, p < 0.001; β = −0.07, p = 0.005). Experience of being bullied was also associated with the level of PWB (β = −0.08, p = 0.001).

**Table 2:** Linear regression analysis of association of sex, internet use, being bullied and deliberate self-harm on adolescents’ level of happiness at age 13 years.

Dependent variable	Independent variable	β	t	p
SAS, 13 yr	Sex	−0.06	−2.76	0.006
	SAS, 12 yr	0.47	20.4	<0.001
	Friend	0.1	4.54	<0.001
	Deliberate self-harm	−0.07	−3.27	0.001
	Internet use in days off school	−0.07	−2.98	0.003
PWB, 13 yr	SAS, 12 yr	0.08	3.5	<0.001
	PWB, 12 yr	0.38	15.93	<0.001
	Friend	0.08	3.21	0.001
	Deliberate self-harm	−0.14	−6.15	<0.001
	Internet use in days off school	−0.07	−2.84	0.005
	Bullied, 13 yr	−0.08	−3.36	0.001

PWB: psychological well-being; SAS: social adaptation status

**Fig 1:**
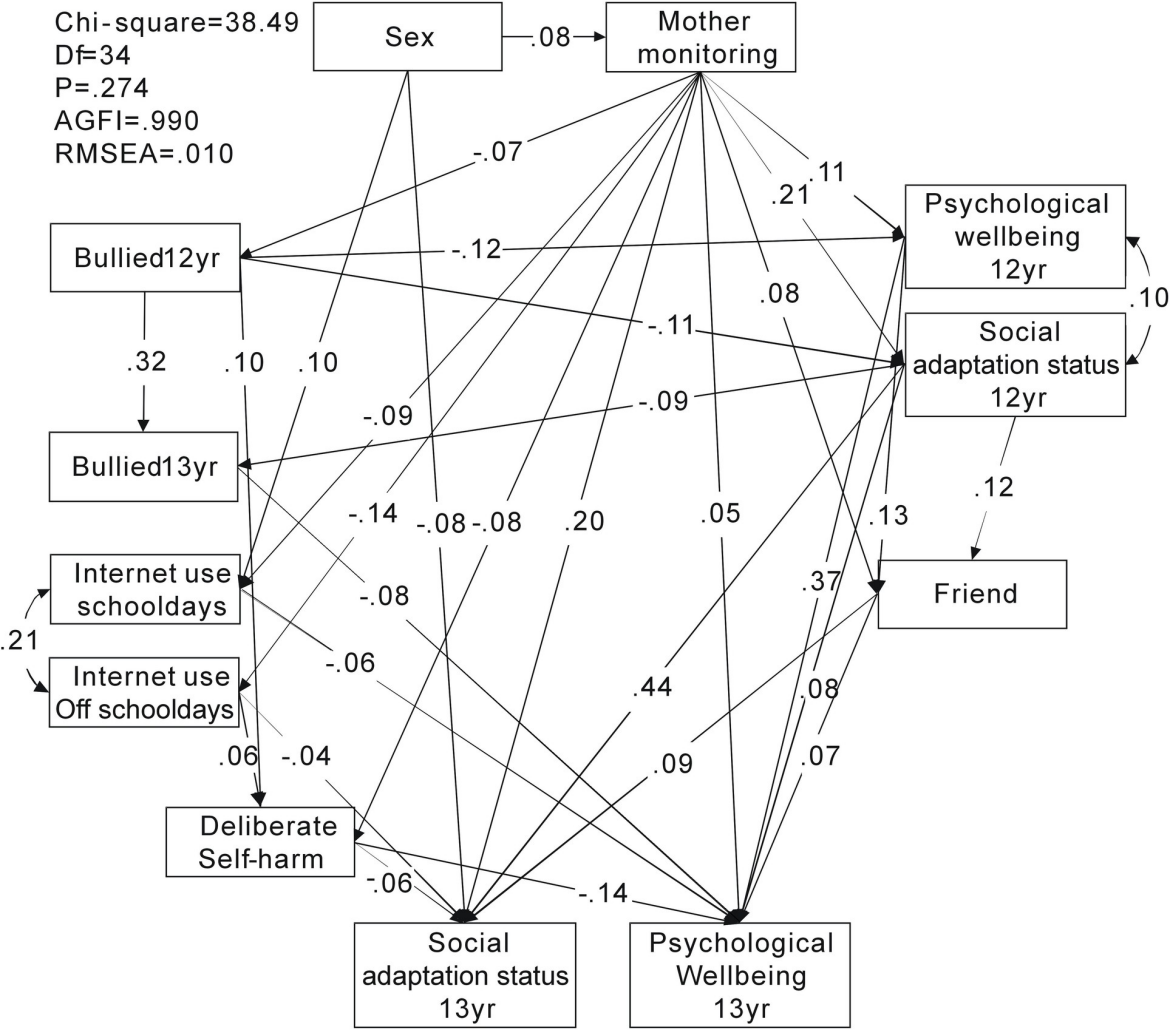
Pathway relationships among maternal monitoring, bullying, internet use, deliberate self-harm and self-perceived happiness. AGFI: adjusted goodness-of-fit; RMSEA: root mean square error of approximation

**Fig 2:**
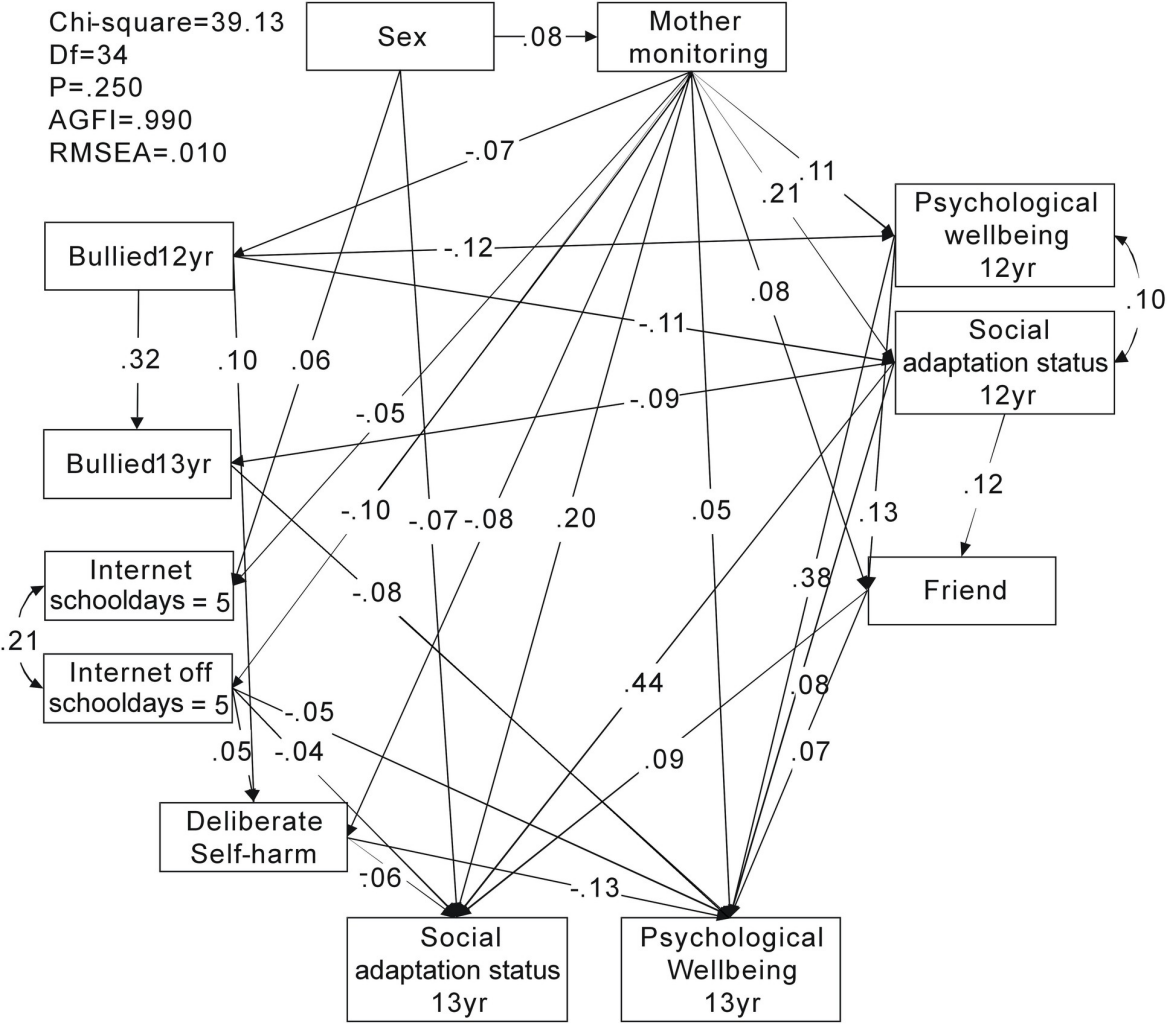
Pathway relationships among maternal monitoring, bullying, internet use >5 h/day, deliberate self-harm and self-perceived happiness. AGFI: adjusted goodness-of-fit; RMSEA: root mean square error of approximation

Two SEMs were analyzed to investigate the pathway relationship of internet use, experience of being bullied, and level of happiness in adolescents. The first model investigated internet use as a continuous variable, and in the second model, internet use was dichotomized into those who spent >5 h/day online and those who did not (dummy variable of those who spent >5 h/day online as 1, and others as 0). The results of the pathway associations in Figs 1 and 2 are presented in Table 3.

**Table 3:** Structural equation modeling of association among maternal monitoring, bullying, internet use, deliberate self-harm and self-perceived happiness (Fig 1), and internet use (Fig 2).

Fig 1	β	p
Sex → SAS, 13 yr	−0.08	<0.001
Sex → internet use school days, 13 yr	0.10	<0.001
Bullied 12 yr → PWB 12 yr	−0.12	<0.001
Bullied 12 yr → SAS 12 yr	−0.11	<0.001
Bullied 12 yr → bullied 13 yr	0.32	<0.001
SAS 12 yr → bullied 13yr	−0.09	<0.001
Maternal monitoring → internet use school days 13 yr	−0.09	<0.001
Maternal monitoring → internet use days off school 13 yr	−0.14	<0.001
Maternal monitoring → deliberate self-harm	−0.08	0.002
Bullied 12 yr → deliberate self-harm	0.10	<0.001
Internet use days off school → deliberate self-harm	0.06	0.036
Maternal monitoring → SAS 13 yr	0.20	<0.001
Maternal monitoring → PWB 13 yr	0.05	0.034
Friends → SAS 13 yr	0.09	<0.001
Friends → PWB 13 yr	0.07	0.003
SAS 12 yr → SAS 13 yr	0.44	<0.001
PWB 12 yr → PWB 13 yr	0.37	<0.001
SAS 12 yr → PWB 13 yr	0.08	0.002
Deliberate self-harm → SAS 13 yr	−0.06	0.007
Deliberate self-harm → PWB 13 yr	−0.14	<0.001
Internet use schooldays → PWB 13 yr	−0.06	0.012
Internet use off-school → SAS 13 yr	−0.04	0.047
Fig 2	β	p
Sex → SAS 13cyr	−0.07	<0.001
Sex → internet use ≥5 h/d school days 13 yr	0.06	<0.001
Bullied 12 yr → PWB 12 yr	−0.12	<0.001
Bullied 12 yr → SAS 12 yr	−0.11	<0.001
Bullied 12 yr → bullied 13 yr	0.32	<0.001
SAS 12 yr → bullied 13 yr	−0.09	<0.001
Mother monitoring → internet use ≥5 h/d school days 13 yr	−0.05	0.043
Maternal monitoring → internet use ≥5 h/d days off school 13 yr	−0.10	<0.001
Maternal monitoring → deliberate self-harm	−0.08	0.002
Bullied 12 yr → deliberate self-harm	0.10	<0.001
Internet use ≥5 h/d days off school → deliberate self-harm	0.05	0.052
Mother monitoring → SAS 13 yr	0.20	<0.001
Mother monitoring → PWB 13 yr	0.05	0.036
Friends → SAS 13 yr	0.09	<0.001
Friends → PWB 13 yr	0.07	0.003
SAS 12 yr → SAS 13 yr	0.44	<0.001
PWB 12 yr → PWB 13 yr	0.38	<0.001
SAS 12yr → PWB 13 yr	0.08	0.002
Deliberate self-harm → SAS 13 yr	−0.06	0.007
Deliberate self-harm → PWB 13 yr	−0.13	<0.001
Internet use ≥5 h/d days off school → SAS 13 yr Internet use ≥5 h/d days off school → PWB 13 yr	−0.04 −0.05	0.045 0.029

PWB: psychological well-being; SAS: social adaptation status

The first model of the pathway relationship of internet use, experience of being bullied, and level of happiness in adolescents at age 12 and 13 years resulted in p = 0.082 (>0.05), adjusted goodness-of-fit index (AGFI) = 0.988 (>0.9), and root mean square error of approximation (RMSEA) = 0.015 (<0.08), implying that the null model approximated the real structure (Fig 1). Our SEM shows a positive correlation between the amount of time adolescents spent online during schooldays and days off school (*r* = 0.21, p < 0.001). This means that those who spent more time online during school days were also more likely to spend more time online on days off school. Girls had better SAS than boys, and spent more time online during schooldays (β = −0.08, p < 0.001; β = 0.10, p < 0.001). Having been bullied at age 12 years was associated with a lower level of happiness at the same age (PWB: β = −0.12, p < 0.001; SAS: β = −0.11, p < 0.001). Those who were bullied and those with lower SAS at age 12 years were more likely to experience bullying at age 13 years (β = 0.32, p < 0.001; β = −0.09, p < 0.001). Factors associated with internet use at age 13 years included sex and maternal monitoring, with adolescents that perceived more maternal monitoring spending less time online (schooldays: β = −0.09, p < 0.001; days off school: β = −0.14, p < 0.001). Perceiving less maternal monitoring, being bullied at age 12 years, and spending more time online during days off school were associated with increased likelihood of deliberate self-harm (β = − 0.08, p = 0.002; β = 0.10, p < 0.001; β = 0.06, p = 0.036). Finally, factors associated with the SAS of adolescents at age 13 years included deliberate self-harm, sex, mother monitoring, level of happiness at age 12 years, and interaction with friends. Those who perceived more maternal monitoring, had more frequent interaction with friends, and perceived a higher level of happiness at age 12 had a higher level of happiness at age 13 years (maternal monitoring: β = 0.20, p < 0.001; β = 0.05, p = 0.034; interaction with friends: β = 0.09, p < 0.001; β = 0.07, p = 0.003; SAS at 12 years: β = 0.44, p < 0.001; PWB at 12 years: β = 0.37, p < 0.001; β = 0.08, p = 0.002). In contrast, those who deliberately harmed themselves had a lower level of happiness (SAS: β = −0.06, p = 0.007; PWB: β = −0.14, p < 0.001). Furthermore, adolescents who spent more time online during schooldays had a lower PWB (β = −0.06, p = 0.012); however, those who spent more time online during days off school had a lower SAS (β = −0.04, p = 0.047).

When internet use was dichotomized into those who spent >5 h/day online and those who did not, the model resulted in p = 0.067 (>0.05), AGFI 0.988 (>0.9), and RMSEA 0.016 (<0.08). This implies that the null model approximated to the real structure (Fig 2). Apart from the pathways found in the previous model, this model found that adolescents who spent >5 h/day online during days off school perceived a lower overall level of happiness at age 13 (SAS: β = −0.04, p = 0.045; PWB: β = −0.05, p = 0.025).

## Discussion

Our national birth cohort pilot study found that at age 12 years, approximately one quarter (24.3%) of adolescents reported having been bullied, with the rate of bullying decreasing to 19.8% at age 13. Furthermore, those who had been bullied at 12 years perceived lower PWB and SAS. Those with a lower SAS at age 12 years were at higher risk of being bullied at 13 years, which indirectly affected their PWB at 13 years. Five percent (5.4%) of 13-year-olds reported deliberate self-harm. SEM showed that those who had been bullied at age 12 years were at greater risk of deliberate self-harm. With regard to the association of internet use and perceived level of happiness, a negative association was found between time of internet use during schooldays and PWB. In contrast, internet use during days off school was negatively associated with SAS. When internet use was dichotomized into those who spent >5 h/day online and those who did not, 6% of adolescents spent >5 h/day online during school days, and 32.7% spent >5 h/day online during days off school. Adolescents who spent >5 h/day online during days off school were at higher risk of deliberate self-harm, and perceived lower levels of happiness (PWB and SAS). These results show that previous experience of bullying and excessive internet use during days off school are risk factors for deliberate self-harm, and lower perceived level of happiness in adolescents aged 13 years.

Among the 1,457 adolescents, 24.3% of the 12-year-olds reported having been bullied, with 19.8% at age 13. This is similar to the rate of 22.4% in the United Kingdom [19], and 26% among 12-year-old Finnish students [20]. The decrease in the rate of bullying from age 12 to 13 years is in line with an international large-scale survey including 40 countries that showed a trend of decreasing prevalence in victimization with increasing age [21]. However, it should be noted that in Taiwan, 12 and 13 years is a transitional period from elementary to middle school. Our results show that, even with the change in school and social structure, those who were bullied in elementary school were still likely to be bullied in middle school. Our study also found that being bullied was negatively associated with overall happiness (PWB and SAS) at age 12 years. However, those with lower level of SAS at age 12 were at higher risk of being bullied at 13, leading to lower PWB. Victims of bullying in Finland and Spain also showed a significant decrease in level of happiness, compared to adolescents who were not bullied [20, 22]. Unfortunately, those with a low level of happiness are also maladapted in school and thus become more susceptible to bullying [23]. The negative cycle between level of happiness and bullying was shown in this follow-up study.

Seventy-nine (5.4%) of the 1,457 13-year-olds in our study reported deliberate self-harm in the previous year. This is lower than the average of 9.5% for 12-month prevalence for deliberate self-harm using a single item question in a review of international studies of adolescents [24]. A similar report of 6.9% was found in a 12-month prevalence among 15–16-year-olds in England [25]. In our study, the SEM showed that being bullied at age 12 years and spending more time online increased the risk of deliberate self-harm at 13. A previous review also found a positive association between bullying and deliberate self-harm [26]. As well as quantitative analysis, quantitative research also supports bullying as a contributing factor to self-harm in school-aged children [27]. Our study found that bullying in elementary school (at age 12 years) was associated with deliberate self-harm in middle school (age 13 years); however, bullying at age 13 years was not associated with deliberate self-harm at that age. A study in Australia found that bullying peaked at the transition period from primary to secondary school [28]. When adolescents transit from elementary to middle school, their social structure changes, and new friendships and social hierarchy also develop [29]; thus adolescents are presented with a new social ecology in middle school. For adolescents who were victimized in elementary school, the new environment is an opportunity to build a new peer network and support. In addition to bullying, longer internet use during days off school was also associated with higher possibility of deliberate self-harm. Previous reviews have also found that high levels of internet use and internet addiction are associated with deliberate self-harm [30].

Using 5 h/day as a cutoff for excessive internet use, 6% of the 13-year-old adolescents reported that they spent >5 h/day online during schooldays, and 32.7% on days off school. The TBCS-P found that 5.3% of the 12-year-olds spent >5 h/day online during schooldays, which is similar to the 6% found in the 13-year-olds. Both of these results are within the range of 2.6–10.9% found in a previous review of internet addiction [31]. We found a negative association between time spent online and perceived level of happiness. Adolescents who spent >5 h/day online during schooldays perceived a lower PWB, and those who spent >5 h/day during days off school perceived a lower overall level of happiness. A meta-analysis of 23 studies across different age groups found a negative correlation between problematic internet use and well-being [32]. A study of Japanese university students also found a similar result [33]. Although internet and social media can increase social interaction with friends and family, Kraut et al. found that this phenomenon is only true for people who were already rich in social capital, and for those who lack interpersonal connections in real life, internet use tends to reinforce social isolation [34]. The negative association between internet use and happiness may only be found in collectivist cultures like Asia, in which happiness is associated with social harmony, as opposed to individualistic cultures which positive hedonic experiences is associated with personal achievement [35].

Besides the risk factors mentioned here, we found that maternal monitoring was a protective factor against being bullied, internet use, and deliberate self-harm. Furthermore, it was also positively associated with level of happiness in 12- and 13-year-old adolescents. This is in line with previous studies showing that parental monitoring and protection are protective factors in preventing adolescents from participation in problematic behaviors [9], including internet addiction [36]. Similarly, a meta-analysis found that positive parenting practices, including warm and affectionate parent–child relationships, good communication, parental supervision, and parental involvement and support are protective factors against either becoming a bully or being a victim of bullying [37].

One limitation of our study was that we measured only the hours adolescents spent online. Although internet addiction is included in the fifth edition of the Diagnostic and Statistical Manual of Mental Disorders, no consensus has been reached on an official diagnostic criteria for internet addiction. Using the concept of compulsive–impulsive spectrum disorder, four components of excessive use, withdrawal, tolerance, and adverse consequences are most commonly included in current measurements of internet addiction [38]. Further information regarding the adverse consequences of excessive internet use may provide us with better understanding of online behavior, and a clearer distinction between internet usage and excessive use. Another limitation was that all information collected was self-reported. Furthermore, many of our variables were collected from single item questions (including experience of being bullied, deliberate self-harm, and friends) and not complete measurements. They were thus prone to measurement error, including reliability and/or stability information bias. Since this study was part of a national birth cohort study, with the aim of collecting all information related to the development and health of these children, only limited information from each aspect can be collected.

Our large sample from the national birth cohort pilot study with follow-up of 12- and 13-year-old adolescents showed that 24.3% of the 12-year-olds reported being bullied, compared with 19.8% at age 13 years. Maternal monitoring was found to reduce the risks of being bullied, excessive internet use, and deliberate self-harm. However, previous studies have found that it is often difficult to implement parental involvement to prevent bullying [39]. Because parents have a pervasive influence on adolescents’ behavior, parenting skills and positive interaction should also be emphasized when addressing adolescents’ bullying, excessive internet use, and deliberate self-harm issues. Furthermore, we found that previous experience of bullying and spending >5 h/day online during days off school were risk factors for deliberate self-harm. Spending >5 h/day online may be a warning sign for excessive internet use. This criterion could therefore be used as an indicator in future preventive action programs to screen out those who are at high risk of excessive internet use, deliberate self-harm, and PWB and SAS issues.
